# Diffusion-Weighted MRI and FDG-PET in Diagnosis of Endometrial Stromal Nodule

**DOI:** 10.1155/2015/540283

**Published:** 2015-01-28

**Authors:** Shunsuke Maruyama, Yukiyasu Sato, Yumiko Satake, Hiroko Mise, Tomoko Kim

**Affiliations:** Department of Obstetrics and Gynecology, Otsu Red Cross Hospital, Japan

## Abstract

Preoperative differentiation of benign endometrial stromal nodule (ESN) from malignant low-grade endometrial sarcoma (LGESS) is challenging, because it requires histological evaluation of the tumor-myometrium interface, which is difficult to obtain in conventional endometrial curettage. A 72-year-old postmenopausal woman presented with 5-year history of persistent vaginal bleeding. Histological examination of the endometrial curettage specimen revealed hyperplasia of apparently normal endometrial stromal cells. T2-weighted magnetic resonance imaging (T2W-MRI) showed polypoid tumor occupying the entire uterine cavity. The tumor exhibited high signal intensity in diffusion-weighted MRI (DW-MRI) and intense accumulation of ^18^F-fluorodeoxyglucose (FDG) in positron emission tomography (PET). Intense FDG accumulation was also observed in the left internal iliac region. Total abdominal hysterectomy, bilateral salpingo-oophorectomy, and pelvic lymphadenectomy were performed under the diagnosis of LGESS with lymph node metastasis. However, postoperative histological examination proved that the tumor was ESN without lymph node metastasis. Since mitotic figure is no longer included in the diagnostic criteria of ESN or LGESS, ESN could exhibit high cellularity and high proliferative activity as observed in this case. Therefore, DW-MRI or FDG-PET is not useful in the differentiation of ESN from LGESS.

## 1. Introduction

Endometrial stromal tumor (EST) of the uterus is a rare neoplasm, whose annual incidence is estimated to be 2 per million [1]. According to the latest World Health Organization classification, EST is divided into three categories: (1) endometrial stromal nodule (ESN), (2) low-grade endometrial stromal sarcoma (LGESS), and (3) undifferentiated endometrial sarcoma (UES) [2].

ESN is a benign tumor that is mainly composed of cells resembling normal proliferative-phase endometrial stromal cells [3]. As long as lobulated or finger-like protrusions into the adjacent myometrium are not >3 mm in the depth and are not >3 in the number and there is no vascular invasion, the tumor is regarded as ESN. More extensive myometrial invasion or the presence of vascular involvement indicates that the tumor is LGESS. Accordingly, precise histological diagnosis of ESN requires evaluation of the tumor-myometrium interface, which is difficult to obtain in conventional endometrial curettage. As a result, for most cases of ESN, hysterectomy is selected as primary treatment and the diagnosis is confirmed only after thorough evaluation of the resected uterus.

LGESS has been reported to exhibit high signal intensity in diffusion-weighted magnetic resonance imaging (DW-MRI) and intense accumulation of ^18^F-fluorodeoxyglucose (FDG) in positron emission tomography (PET) [4–6]. Since most of benign uterine fibroids exhibit low signal intensity in DW-MRI and lack significant FDG accumulation in FDG-PET, these modalities are considered useful in preoperative differentiation of uterine fibroid from LGESS. To date, however, there has been no report on the finding of ESN in DW-MRI or FDG-PET. Thus, it is unclear whether DW-MRI or FDG-PET is similarly useful in distinguishing ESN from LGESS.

Here, we present a case of ESN that exhibited high signal intensity in DW-MRI and intense FDG accumulation in FDG-PET. The cells resembling normal endometrial stromal cells were densely distributed in the endometrial curettage specimens. These findings led to the misdiagnosis of LGESS and total abdominal hysterectomy and bilateral salpingo-oophorectomy with pelvic lymphadenectomy were performed. Postoperative histological evaluation proved that the tumor was ESN without lymph node metastasis.

## 2. Case Presentation

**Figure 1:**
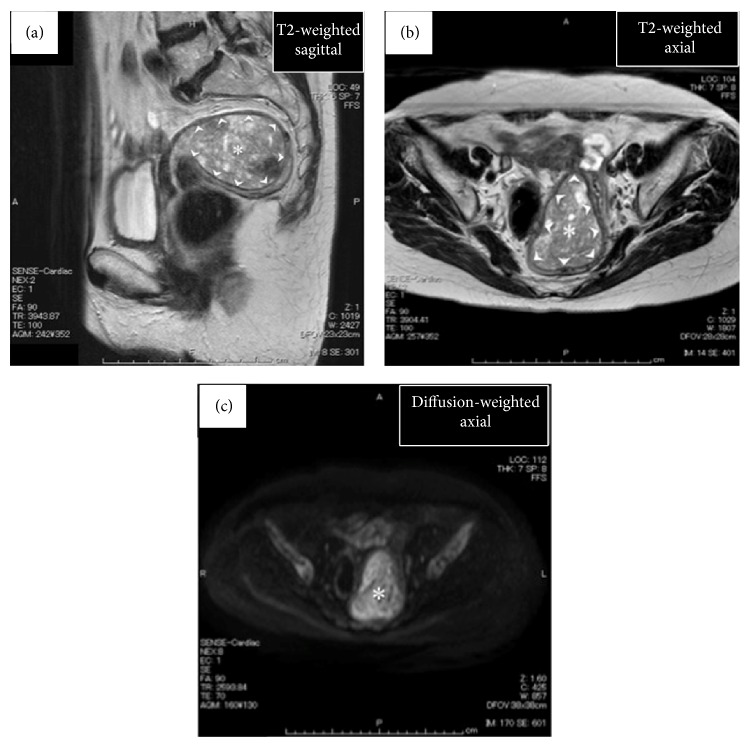
Findings of pelvic magnetic resonance images (MRI). ((a) and (b)) Sagittal (a) and axial (b) planes in T2-weighted MRI. Uterine cavity is filled with heterogeneous polypoid tumor (∗). Note the smooth boundary between the tumor and the thinned myometrium (arrowheads). (c) Axial plane in diffusion-weighted MRI. Uterine tumor (∗) exhibits high signal intensity as compared with the surrounding myometrium.

**Figure 2:**
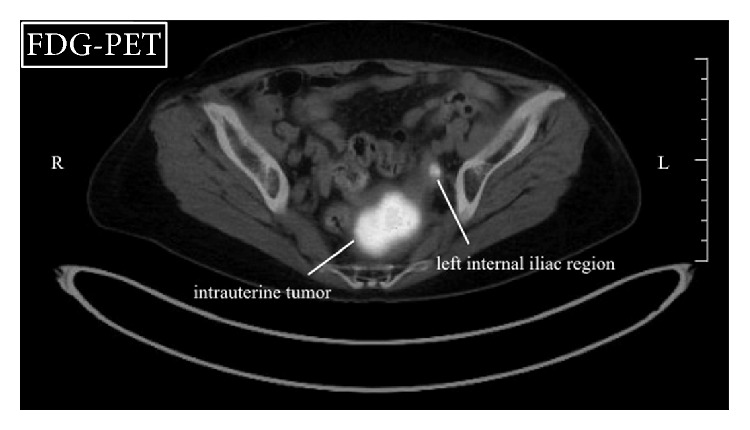
Findings of FDG-PET. Intense accumulation of FDG is observed not only in the uterine tumor but also in the left internal iliac region.

**Figure 3:**
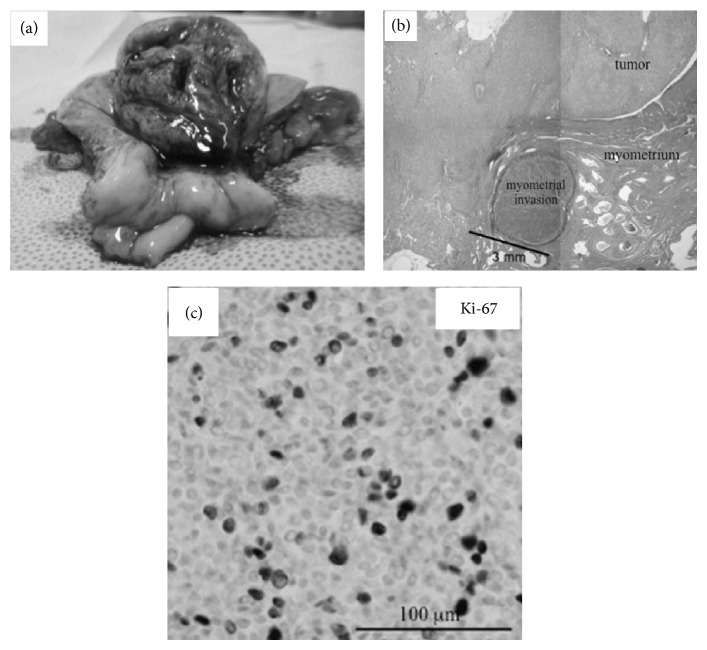
Macroscopic and microscopic findings of the uterine tumor. (a) Macroscopic appearance of the uterine tumor. Uterus is opened by longitudinal incision through the anterior wall. Note that huge polypoid tumor grows from posterior wall of the uterus. The tumor is connected to the posterior myometrium with wide pedicle. (b) Microscopic finding in low-power field (HE staining). One finger-like projection is protruded into the myometrium. Note that the length between the tumor-myometrium interface and the tip of the tumor protrusion does not exceed 3 mm. (c) Microscopic finding in high-power field (immunostaining with Ki-67 antibody). Note that numerous tumor cells are positive for Ki-67, a cell proliferation marker. On average, mitotic activity is estimated to be 3 mitotic figures/10 high-power fields.

A 72-year-old, para 2, gravida 3, postmenopausal woman (menopause at the age of 52) consulted our hospital due to abnormal vaginal bleeding persistent for 5 years. Her final Pap smear test at the age of 66 had been negative. On the vaginal inspection, large blood clots were retained in the vaginal cavity with small amount of bleeding still continuing from the cervical os. Bimanual pelvic examination showed retroflexed uterus that was enlarged to man's fist size. Transvaginal ultrasonography delineated heterogeneous endometrium thickened to 24.7 mm. The boundary between the endometrium and the myometrium appeared smooth. Both Pap smear test and endometrial cytology proved to be negative. Laboratory data revealed moderate anemia (hemoglobin: 8.2 g/dL). The examined tumor markers were all within the normal ranges (CA19-9: 7.2 U/mL, CA125: 8.4 U/mL, CEA: 1.4 ng/mL, and SCC: 0.7 ng/mL). Microscopically, uniform small cells with scanty cytoplasm and oval nuclei that resembled normal endometrial stromal cells were densely distributed in the endometrial curettage specimens. Since the tumor-myometrium interface was not contained in the specimens, the tentative pathological diagnosis was LGESS or ESN. T2-weighted magnetic resonance imaging (T2W-MRI) delineated polypoid tumor occupying the entire uterine cavity. Boundary between the tumor and the thinned myometrium appeared smooth, but minimal invasion into the myometrium could not be ruled out (Figures 1(a) and 1(b)). In DW-MRI, the tumor exhibited high signal intensity (Figure 1(c)). In FDG-PET, intense FDG accumulation was observed not only in the uterine tumor but also in the left internal iliac region (Figure 2), suggesting that the uterine tumor was malignant and was metastasized to the left internal iliac lymph node. Collectively, preoperative diagnosis of LGESS with lymph node metastasis was made. Total abdominal hysterectomy, bilateral salpingo-oophorectomy, and pelvic lymphadenectomy were performed. At laparotomy, serosal surface of the uterus was intact and no dissemination was detected in the abdominal cavity. None of the dissected lymph nodes were abnormally swollen. Macroscopically, polypoid mass occupying the entire uterine cavity was connected to the posterior uterine wall with a wide pedicle (Figure 3(a)). Microscopically, the uterine mass was composed of the cells that closely resembled normal endometrial stromal cells. Thorough histological examination of the pedicle demonstrated one finger-like projection from the tumor into the myometrium that did not exceed 3 mm in the depth (Figure 3(b)). No tumor cell was detected inside the myometrial vessels or in the dissected lymph nodes. Based upon these findings, definitive diagnosis of ESN was made. In immunostaining, numerous tumor cells (3 cells per 10 high-power fields on average) were positive for a proliferation marker, Ki-67, indicating relatively high proliferative activity of the tumor (Figure 3(c)).

No postoperative adjuvant therapy was offered. The patient has been followed up in the outpatient clinic without any sign of recurrence as of one year after the surgery.

## 3. Discussion

Preoperative differentiation between ESN and LGESS by histological examination of the specimens obtained from conventional endometrial curettage is extremely difficult, because the tumor-myometrium interface is unlikely to be contained in these specimens [7]. Although ultrasonography, T2W-MRI, and gadolinium-enhanced T1-weighted MRI can delineate the tumor-myometrium interface, it is often difficult to rule out the myometrial invasion, especially when the myometrium is thinned by the intrauterine mass and/or the invasion is confined to the shallow portion of the myometrium.

DW-MRI and FDG-PET have become widely recognized as powerful tools to differentiate malignant from benign tumor. In DW-MRI, signal intensity is inversely related to the degree of microscopic water diffusion in the tissue, which is mainly restricted by the high cellularity. FDG-PET visualizes the accumulation sites of FDG (a glucose analogue) that was systemically administered. The cells with high metabolic and/or high proliferative activity predominantly take up FDG. Since most of malignant tumors have high cellularity and are composed of highly proliferative cells, they generally exhibit high signal intensity in DW-MRI and intense FDG accumulation in FDG-PET.

Like other malignant tumors, LGESS has been demonstrated to show high signal intensity in DW-MRI and high FDG accumulation in FDG-PET [4–6]. Since most of uterine fibroids exhibit low signal intensity in DW-MRI and lack significant FDG accumulation in FDG-PET, these modalities are considered useful in preoperative differentiation of uterine fibroids from LGESS. In the present case, however, the uterine tumor, which postoperatively proved to be benign ESN, exhibits high signal intensity in DW-MRI and intense FDG accumulation in FDG-PET, both of which are reminiscent of malignant tumor. Together with the finding that internal iliac region also accumulated FDG, misdiagnosis of LGESS with lymph node metastasis was made. The FDG-PET was carefully reviewed after the surgery, reaching to the conclusion that the extrauterine FDG accumulation corresponded to the urine transiently retained in the left ureter rather than the lymph node.

Consistent with the findings observed in DW-MRI and FDG-PET, histological examination of the present ESN demonstrated high cellularity with frequent mitotic figures as indicated by numerous Ki-67-positive cells (3 mitotic figures/10 high-power fields). Since it has been demonstrated that proliferative activity of EST does not influence the prognosis [8], mitotic figure is no longer considered important for the differential diagnosis of EST [7, 9]. Thus, it is not surprising that some of ESNs have high proliferative activity, leading to high signal intensity in DW-MRI and intense FDG accumulation in FDG-PET, as seen in this case. In this respect, DW-MRI or FDG-PET is not useful for differentiation of ESN from LGESS and histological evaluation of tumor-myometrium interface is still indispensable for the diagnosis of ESN.

In conclusion, we here present a case of benign ESN that exhibited high signal intensity in DW-MRI and intense FDG accumulation in FDG-PET, both of which are characteristic features for malignant tumors. These findings are not surprising because mitotic figure in the tumor is not included in the diagnostic criteria of ESN or LGESS. Therefore, DW-MRI or FDG-PET is not useful in the differentiation of ESN from LGESS, which, for the present, only can be made by histological evaluation of the tumor-myometrium interface.
